# Increase in new-onset type 1 diabetes diagnoses among Brazilian children and adolescents during the COVID-19 pandemic

**DOI:** 10.1016/j.jped.2025.04.001

**Published:** 2025-05-29

**Authors:** Renata Szundy Berardo, Melanie Rodacki, Bruna S. Pugliese, Fernanda Roisman, Juliane Rocha, Daniel Gilban, Bianca Balassiano, Cristine Barboza Beltrão, Isla Aguiar Paiva, Juliana Veiga Moreira, Jorge Luescher, Ludmila Campos, Isabella Coutinho, Isabel Rey Madeira, Alessandra S.M.F. da Costa, Lucianne R.M. Tannus, Nádia C.P. Rodrigues, Lenita Zajdenverg

**Affiliations:** a Hospital Federal dos Servidores do Estado (HFSE) do Rio de Janeiro, Rio de Janeiro, RJ, Brazil; b Universidade Federal do Rio de Janeiro (UFRJ), Hospital Universitário Clementino Fraga Filho. Departamento de Medicina Interna, Seção de Nutrologia e Diabetes, Rio de Janeiro, RJ, Brazil; c Hospital Geral de Bonsucesso (HGB), Rio de Janeiro, RJ, Brazil; d Instituto Estadual de Diabetes e Endocrinologia Luiz Capriglione (IEDE), Rio de Janeiro, RJ, Brazil; e Universidade Federal do Rio de Janeiro (UFRJ), Instituto de Puericultura e Pediatria Martagão Gesteira (IPPMG), Rio de Janeiro, RJ, Brazil; f Universidade Estadual do Rio de Janeiro (UERJ), Policlínica Piquet Carneiro, Rio de Janeiro, RJ, Brazil

**Keywords:** T1dm, Covid-19, Ketoacidosis, Epidemiology, T1DM

## Abstract

**Objective:**

This study aimed to determine if there was a rise in new T1DM cases in children during the pandemic in a large metropolitan area in Brazil.

**Methods:**

The authors conducted a cross-sectional study at five public tertiary care centers that specialize in diabetes in children, comparing all new T1DM cases (ages 0.5–18y) diagnosed from March 2020 to December 2021 (pandemic period, PP) with those from March 2018 to December 2019 (historical period, HP).

**Results:**

There were 167 new cases in the PP compared to 99 in the HP, reflecting a 68.7 % rise, with a notable peak observed in the third quarter of 2020 (*p* = 0.006). The average age of diagnosis was 8.4 ± 4.2 years in the PP and 7.5 ± 3.6 years in the HP, with no significant difference (*p* = 0.06). The gender distribution, BMI Z scores, and duration of diabetes symptoms before diagnosis were similar. The incidence of diabetic ketoacidosis (DKA) at onset was elevated but did not increase during the pandemic (62.6 % historical vs. 59.3 % pandemic period). During the PP, 24 % of patients reported symptoms of SARS-CoV-2 infection before the diagnosis of T1DM or at admission, and 13 % (7/53) of tested patients were positive for SARS-CoV-2.

**Conclusions:**

The present findings indicate a significant rise in new T1DM cases among children during the COVID-19 pandemic compared to prior years, without differences in DKA at onset.

## Introduction

Since December 2019, the world has been challenged by the coronavirus disease (COVID-19), which was labeled a global pandemic on March 11, 2020.

In the following years, the understanding of this disease improved. Vaccinations in many countries have progressed, reducing the incidence and severity of the disease. However, long-term consequences have emerged, but the underlying mechanisms remain largely unexplored.

In May 2023, the CDC reported 15,594,079 total child COVID-19 cases, accounting for 17.9 % of all COVID-19 cases, with an overall rate of 20,718 cases per 100,000 children in the population [1].

The first confirmed case of COVID-19 in Brazil was reported on February 25, 2020. Since then, Brazil has recorded over 34 million cases and 688,000 deaths, making it the fifth highest worldwide.

Rio de Janeiro had the second-largest number of cases in the country, experiencing five virus waves: the B1 variant (April-May 2020), Zeta variant (November 2020-January 2021), Gamma variant (February-June 2021), Delta variant (August 2021), and the Omicron variant (December 2021-January 2022) [2].

Vaccination in Brazil began in January 2021 for selected adult groups and was expanded to adolescents aged 12–17 in September 2021.

The official reports significantly underestimated the incidence of SARS-CoV-2 infection. Brazil ranked 125th globally in COVID-19 testing as of October 21, and testing was limited, especially in children, as they often displayed mild or no symptoms [3].

SARS-CoV-2 interacts with host cells by binding to the angiotensin-converting enzyme 2 (ACE2) on cell membranes through its spike protein. ACE2 is found in many glands and organs with endocrine functions. Although COVID-19 typically has a less severe impact on children compared to adults, in some cases, it can lead to a systemic response due to abnormal hyperinflammation, known as multisystem inflammatory syndrome in children (MIS-C), associated with high mortality and apparent involvement of the immune system [4,5].

T1DM is an autoimmune disease potentially triggered by viral infections [6]. Studies have explored the link between the COVID-19 pandemic and the onset of T1DM in children, some reporting an increased incidence of T1DM and severe diabetic ketoacidosis (DKA), while others found no association [7, 8, 9]. A meta-analysis indicated a significant rise in childhood new-onset T1DM, DKA, and higher mean HbA1c levels during the first year of the pandemic compared to pre-pandemic [10]. A recent cohort from the TEDDY study indicated that COVID-19 did not trigger islet-autoantibody positivity in these genetically at-risk children [11]. Contradicting results were found in a similar cohort in which SARS-CoV-2 infection was temporally associated with the development of islet autoantibodies [12].

The incidence of T1DM has been rising globally, including in Brazil. However, the pandemic’s impact on this trend remains uncertain. Given Brazil's high number of COVID-19 cases and the lack of data on new diabetes cases, there is a pressing need for further research [13, 14, 15].

Healthcare in Brazil is provided by the Unified Health System (SUS) and a supplementary system, with over 70 % of the population using the public system for free care. After diabetes diagnosis, children are referred to specialized tertiary care centers, which are crucial for pediatric T1DM treatment in Rio de Janeiro, serving around 6.7 million people as of the 2022 census.

This study aims to compare the number of new T1DM cases between 2018–2019 (historical period) and 2020–2021 (pandemic period) at five pediatric tertiary care centers in Rio de Janeiro to assess the impact of the pandemic.

## Materials and methods

A multicenter, cross-sectional study compared pediatric patients with new-onset T1DM during two periods: March 2020 to December 2021 (PP - pandemic period) and March 2018 to December 2019 (HP - historical period). Data was collected at five centers in Brazil's public healthcare system specializing in pediatric T1DM in metropolitan Rio de Janeiro.

The study included children and adolescents (ages 0.5–18 years) with a new diagnosis of T1DM within the past six months, as determined by glycemic criteria and the need for insulin, according to the ISPAD guidelines. Children under six months of age were excluded to prevent misdiagnosing neonatal diabetes [16].

Clinical data were collected from patient medical records, including age, gender, age at diabetes diagnosis, duration of diabetes symptoms before diagnosis, and body weight, height, and BMI from the first outpatient visit. Informed consent was obtained from legal guardians and patients. The study was approved by the Institutional Ethics Committee and registered on Plataforma Brasil. (protocol number 000662).

During the pandemic, interviews gathered medical histories on COVID-19 symptoms, including infection dates and test results (RT-PCR or IgM antibodies), before the diagnosis of T1DM.

The pandemic group was divided into confirmed COVID-19 (positive RT-PCR or IgM/IgG antibodies), suspected (symptoms of COVID-19, without available or positive testing), - Table 1 and neither [4].

**Table 1:** Clinical manifestations of COVID‐19 infection in children.

Fever
Cough / Nasal symptoms
Pharyngeal erythema / sore throat
Disturbances of smell or taste
Gastrointestinal symptoms (diarrhea, nausea, vomiting, and abdominal pain)
Headache and malaise
Rash
Conjunctivitis
SARS / Respiratory failure
Pediatric inflammatory multisystem syndrome (PIMs)

### Statistical analysis

The data were analyzed using Statistical Package R Project version 4.2.0. Descriptive statistics are presented as absolute and relative frequencies for categorical variables and mean and standard deviation for quantitative variables. Differences in the frequencies of categorical variables were analyzed using a chi-square or Fisher’s exact test. A multinomial model, specifically multinomial logistic regression, was employed to assess differences between groups. The statistical tests applied included maximum likelihood estimation (MLE), Wald tests for the significance of individual coefficients, likelihood ratio tests for model comparison, and the use of confidence intervals to assess the precision of the estimates. The reference groups were the year of diabetes diagnosis (2018) and the 2nd quarter. The year of diagnosis was used as the response variable, and the trimester was used as the explanatory variable.

Student's *t*-test and ANOVA were used to analyze the differences between groups for quantitative variables. The level of statistical significance (α) was *p* < 0.05.

## Results

### Clinical characteristics and demographics

A total of 300 children were diagnosed with T1DM from 2018 to 2021. During the pandemic period (March 2020 to December 2021), 167 cases were reported, compared to 99 cases during the historical period (March 2018 to December 2019), representing a 68.7 % increase. This analysis excludes patients from the first quarter of 2018 and 2020.

The average age was 8.12 years, with a median of 8.16 years (range 0.57–18.4 years). Although the pandemic group was slightly older, this difference was not statistically significant. During the pandemic, ages ranged from 0.57 to 18.4 years, with first and third quartiles at 5.04 and 11.15 years, respectively. In the historical period, ages ranged from 1 to 15.9 years, with first and third quartiles at 4.63 and 9.8 years.

There was no significant difference in gender distribution or BMI Z scores between the groups (Table 2).

**Table 2:** Demographic and clinical characteristics of historical versus pandemic period patients^#^ [1].

	Historical period	Pandemic period	
	2018–2019	2020–2021	*P*-value
N patients	99	167	
Average age (years) -mean (SD)	7.5 (3.6)	8.4 (4.2)	0.06
Z-score BMI - mean (SD)	0.3 (1.2)	0.2 (1.2)	0.79
Female sex - *n* (%)	49 (49.5)	80 (47.9)	0.90
Ketoacidosis (yes) - *n* (%)	62 (62.6)	99 (59.3)	0.99
Duration of T1DM symptoms before diagnosis - days avg (SD)	28.81 (26.77)	28 (32.43)	0.83

#Excluded first quarter of 2018 and 2020.

### Diabetic ketoacidosis and symptoms of diabetes

DKA frequency (62.6 % HP vs. 59.3 % PP) and the average duration of classic symptoms (polyuria, polydipsia, nocturia, enuresis, weight loss) before the diagnosis of diabetes (28.8 days historical vs. 28 days pandemic) were similar between groups.

### Seasonal distribution of new T1DM diagnoses

**Figure 1:**
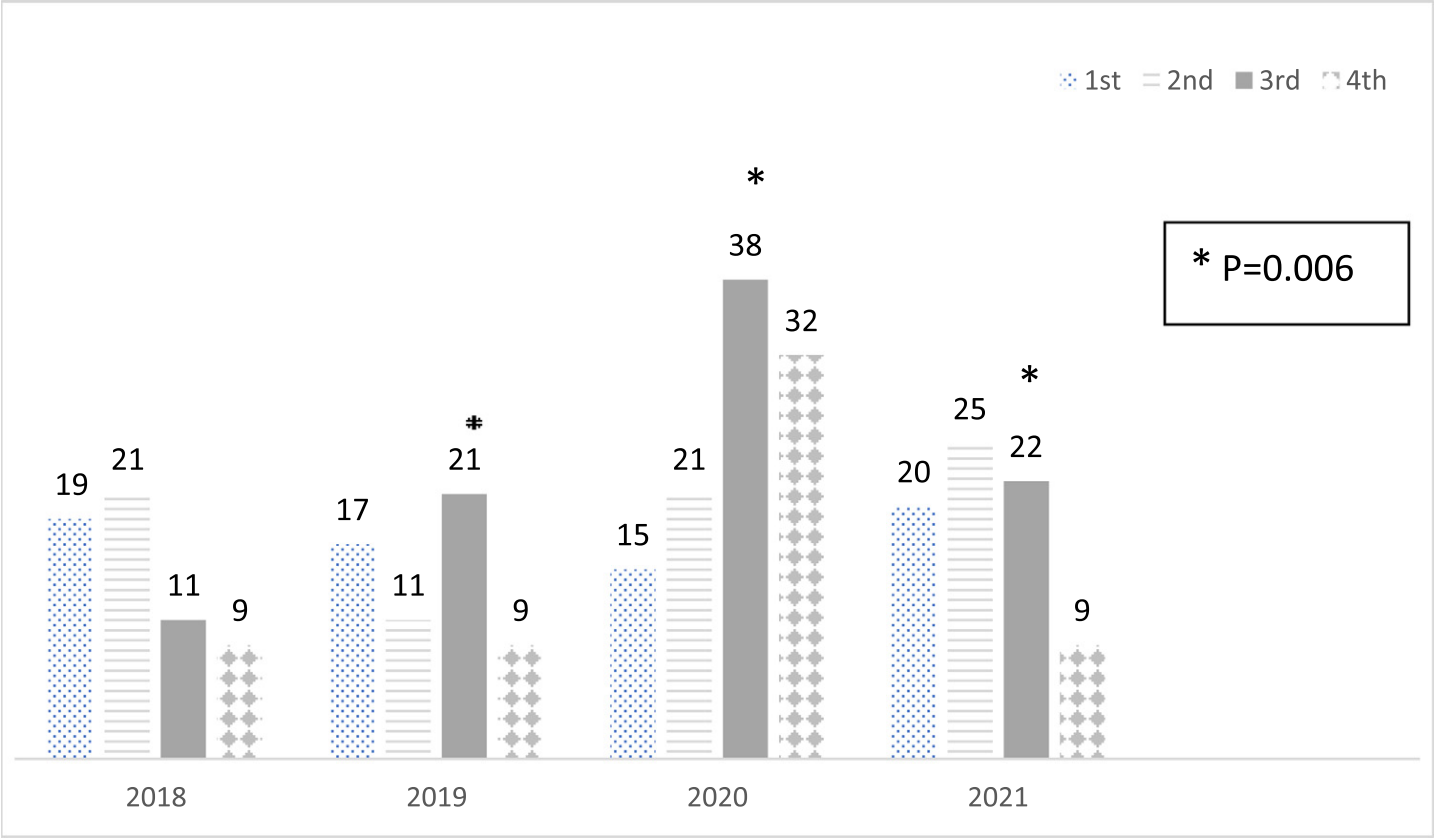
Distribution of new cases of diabetes each year and quarter.

The distribution of diagnoses by quarter and year is illustrated in Figure 1. A significant difference was noted in the distribution of diagnoses across the quarters each year, with a notable increase in 2020. Specifically, there was a substantial increase in the number of new cases of T1DM in the third quarter (Q3) of 2020 compared to 2018 and 2019 (*p* = 0.006). Additionally, in the fourth quarter (Q4), there was a significant increase in new T1DM cases between 2018 and 2020. However, this trend did not continue in 2021.

### SARS-CoV-2 infection symptoms and testing

During the pandemic, 40 out of 167 patients (23.9 %) reported symptoms of SARS-CoV-2 infection prior to T1DM diagnosis, and 20 within two weeks. The average time from suspicion of SARS-CoV-2 infection to diagnosis of diabetes was 38 days (range: 0 to 487 days). The most frequently reported symptoms were abdominal pain (9.63 %), somnolence (8.43 %), cough, and shortness of breath (7.22 % each). Fever, sore throat, and headache were reported in 9 patients each (5.42 %). Six patients (3.6 %) reported ageusia, and 2 reported skin rash.

Of the symptomatic patients, 5 (8 %) had a positive test result, 17 (42.5 %) tested negative, and 18 were not tested. In three cases, the SARS-CoV-2 antibody status was undetermined, and one result was positive for IgG antibodies. Three patients had positive SARS-CoV-2 RT-PCR concurrent with T1DM diagnosis but were asymptomatic.

None of the patients had been vaccinated at the time of the study.

## Discussion

The present study is the first to examine the effects of the COVID-19 pandemic on new cases of T1DM in children across Latin America, revealing a significant 68.7 % increase in cases. While the incidence of T1DM in Brazil has been rising, a national registry for comparison is lacking. However, a study conducted in another city in the Brazilian urban area indicates an annual increase of 3.1 % in cases among children under 14 years old from 1986 to 2015. Thus, the pandemic-related increase in new cases is significantly higher than expected.

There is growing evidence that SARS-CoV-2 infection is linked to higher rates of diabetes. A meta-analysis found a 66 % increase in new-onset diabetes cases following infection [17], and an excess case above control of 11 to 276 % was found in another review [18]. Another meta-analysis of T1DM in youths revealed a higher incidence rate during the first year of the pandemic [19]. In a study evaluating children, there was an increased risk of a new diagnosis of T1DM at 1, 3, and 6 months after SARS-CoV-2 infection, compared with other respiratory infections [20]. A study using DPV data demonstrated an excess number of new cases of diabetes compared to expected and also found a correlation between the peaks of new cases of diabetes following the waves of COVID-19 in Germany [8]. The Sweet registry analyzed 17,280 T1DM cases from 2018 to 2021 and observed a rise in T1DM incidence across all 92 centers, although within the expected range of the regression line. Conversely, a nationwide study in France failed to demonstrate increased incidence [21,22].

Multiple explanations exist for the increased frequency of T1DM cases during the pandemic years, including direct SARS-CoV-2 action on pancreatic beta cells, severe illness, psychological stress during isolation, obesogenic factors, and other yet unknown factors. Discrepancies between studies in different populations may arise from variations in T1DM incidence trends, genetic risk, timing and intensity of COVID-19 exposure, and differing containment measures [23].

COVID-19 had a severe impact on Brazil, with the infection rate in children likely underestimated due to insufficient testing. Lockdowns were implemented and public schools were closed for an extended period during the pandemic; however, in low-income, densely populated areas of Rio de Janeiro, children and their parents had close social contact with others, including those in the workforce. Additionally, vaccination in Brazil began in January 2021 for adults and expanded to adolescents aged 12 to 17 in September 2021, leaving them unprotected from COVID-19.

None of the patients were vaccinated at the time of their diabetes diagnosis, indicating that vaccination did not trigger autoimmunity in this study.

In contrast to other reports, the authors did not observe an increase in DKA frequency at diagnosis during the pandemic. DKA rates are high in Brazil, with an incidence of 42.3 % in a national study involving 3591 patients (median age 19), 32.8 % in a survey of 545 individuals (median age 11.98) from the studied region, and 58.8 % in 274 patients (median age 7.8). There is an inverse relationship between age at diagnosis and DKA, which may explain the rates observed in this study, with a median age of 8.12 at T1DM diagnosis, which is younger than the global epidemiology average [24, 25, 26, 27, 28, 29, 30]. The disparity in these results may be due to the notably high baseline incidence of DKA in the studied population, at 62.6 %, compared to 15 % in a previous Brazilian study and 24.5 % in Germany, which increased to 34 % and 44.7 %, respectively, which yet remained lower than the incidence in the studied population.

Brazil's testing for SARS-CoV-2 was insufficient, with restricted availability until 2021. Given the scarcity of tests when data collection began, the authors aimed to collect data on SARS-CoV-2 symptoms to estimate underreported infections and identify clinical characteristics. Symptoms of SARS-CoV-2 infection were reported in 24 % of patients, and 13 % of the 53 patients tested had a positive SARS-CoV-2 result before the diagnosis of diabetes. Three patients had no symptoms of SARS-CoV-2 infection but had a detectable RT-PCR for SARS-CoV-2 at admission for T1DM. A similar finding was observed in a cohort of infants, where six patients developed islet antibodies simultaneously with SARS-CoV-2 antibodies, and six others did so at their next visit after testing positive for SARS-CoV-2 [12,17].

The average time from the onset of SARS-CoV-2 symptoms to a diagnosis of diabetes was 38 days. This aligns with CDC findings of diabetes risk within three months post-COVID-19 infection and reports of increased T1DM cases in Germany following each COVID-19 wave [20,30].

The authors could not assess islet antibodies. Therefore, the authors cannot establish any causal or temporal associations between SARS-CoV-2 infection and the increase in autoimmunity. The literature presents conflicting results. A meta-analysis demonstrated an adjusted hazard ratio (HR) of 3.5 for the development of islet autoantibodies in individuals who tested positive for SARS-CoV-2, particularly those under 18 months of age [17]. In contrast, children from the TEDDY study were systematically tested for SARS-CoV-2, and did not show that COVID-19 increased T1DM antibodies [11].

The authors found a significant difference in the frequency of new T1DM cases in the third quarter of 2020 compared to 2018 and 2019, as well as between the quarters of 2020. Several studies suggest a shift in seasonal variation in the presentation of T1DM during the pandemic [22]. The typical seasonality of more cases during the winter season was delayed in Europe and North America following the lockdown early in 2020, with a peak during the summer and autumn months, returning to the previous pattern in 2021. T1DM in Brazil exhibits no clear seasonality, with no significant patterns observed before or after the pandemic. The peak of new T1DM cases in this study occurred in the 3rd quarter of 2020, shortly after the highest COVID-19 hospitalization rates in June and July 2020.

### Strengths and limitations

The present study contributes by providing evidence of the increased frequency of new diabetes cases during the pandemic in a region where data is scarce. The data were collected from multiple centers representative of the population of the metropolitan area, and all observed an increase in the number of children diagnosed with T1DM during the pandemic, which makes referral biases unlikely.

This study is not population-based; therefore, the authors were unable to calculate the incidence rate of pediatric T1DM. Additionally, the authors do not have access to national registries for T1DM in Brazil. Although the time frame might have been too short to exclude the cyclical variation in T1DM incidence described in Europe (20), the use of retrospective data without formal registries could increase bias in diabetes case notifications.

The absence of SARS-CoV-2 serologic testing at the time of T1DM does not allow us to infer how many patients during the pandemic period had COVID-19. However, the clinical data on COVID-19-related symptoms was important in this context because there was a decline in other respiratory infectious diseases in children during the pandemic; therefore, most symptomatic children were likely to have COVID-19.^2^ This study also highlights the clinical characteristics of cases diagnosed during the pandemic and compares them to those from previous years. Furthermore, although the authors did not perform diabetes antibody testing, all patients diagnosed during the pandemic were followed up for an average of three months after enrollment, which helped exclude transient cases of hyperglycemia or misclassification.

There was an increase in the number of cases of T1DM in children and adolescents during the COVID-19 pandemic period in a large metropolitan area population in Brazil. The authors observed a high frequency of DKA in the studied population, similar to the frequency during the pandemic period. This data should raise awareness of T1DM diagnosis in children, which could help prevent DKA, and highlight the need to allocate more resources for diabetes care in the context of an increasing frequency of cases.

## Author contributions

**RSB** was responsible for conceptualization, coordination, data collection, analysis, writing, and editing. **MR** and **LZ** were responsible for conceptualization (lead), writing the original draft (lead), review, and editing. **NR** was responsible for the methodology and formal data analysis. **BP** and **FR** led the software and data collection. **JR, DG, BB, CBB, IAP, JVM, JL, LC, IC, IRM,** and **LT** provided support for conceptualization and data collection and contributed to the original draft, review, and editing equally.

## Declaration of generative AI and AI-Assisted technologies in the writing process

During the preparation of this work, the authors utilized Grammarly AI prompts to verify the text's correctness and readability. After using this tool, the authors reviewed and edited the content as necessary and took full responsibility for the final publication.

## Funding

The author(s) received no financial support for this article's research, authorship, and/or publication.

## Article information

The data supporting this study's findings are available from the corresponding author upon reasonable request.

## Conflicts of interest

The authors declare no conflicts of interest.
